# Riboflavin transporter deficiency, the search for the undiagnosed: a retrospective data mining study

**DOI:** 10.1186/s13023-024-03428-y

**Published:** 2024-11-01

**Authors:** B. Jaeger, E. Hoytema van Konijnenburg, M. A. Groenveld, M. Langeveld, N. I. Wolf, A. M. Bosch

**Affiliations:** 1 Department of Child Neurology, Emma Children’s Hospital, Amsterdam University Medical Centers, Amsterdam, The Netherlands; 2 Department of Metabolic Diseases, Wilhelmina Children’s Hospital, University Medical Center Utrecht, Utrecht, The Netherlands; 3 Department of Neurology, Amsterdam Neuroscience, Amsterdam University Medical Centers, Amsterdam, The Netherlands; 4 Department of Endocrinology and Metabolism, Amsterdam University Medical Centers, Amsterdam, The Netherlands; 5 Department of Child Neurology, Emma Children’s Hospital, and Amsterdam Neuroscience, Cellular and Molecular Mechanisms, Vrije Universiteit, Amsterdam, The Netherlands; 6 Department of Pediatrics, Division of Metabolic Disorders, Emma Children’s Hospital, Gastroenterology, Endocrinology and Metabolism, Amsterdam University Medical Centers, Amsterdam, The Netherlands

**Keywords:** Vitamin B2, Riboflavin transporter deficiency, Neurodegenerative disease

## Abstract

**Background:**

Riboflavin transporter deficiency (RTD) is an inborn error of riboflavin transport causing progressive neurological symptoms if left untreated. While infants with symptomatic RTD rapidly deteriorate, presentation later in childhood or in adulthood is more gradual. Symptoms overlap with more common diseases, carrying a risk of misdiagnosis, and given the relatively recent discovery of the genetic basis of RTD in 2010 it is likely that older patients have not been tested. Treatment with oral riboflavin (vitamin B2) halts disease progression and can be lifesaving. We hypothesized that patients may have been left unrecognized at the time of presentation and therefore we performed a datamining study to detect undiagnosed RTD patients in a tertiary referral hospital.

**Methods:**

A systematic search in Electronic Health Records (EHR) of all patients visiting the Amsterdam University Medical Centers between January 2004 and July 2021 was performed by a medical data text-mining tool. Pseudonymized patient records, matching pre-defined search terms (hearing loss or auditory neuropathy spectrum disorders combined with key clinical symptoms or riboflavin) were screened and included if no definitive alternative diagnosis for symptoms indicating possible RTD was found. Included patients were offered genetic testing. We documented total number of patients with possible RTD, number of patients that underwent genetic testing for RTD and results of genetic testing.

**Results:**

EHR of 2.288.901 patients were automatically screened. Thirteen patients with possible RTD were identified and offered genetic testing. Seven patients chose not to participate. Genetic testing was performed in 6 patients and was negative. The datamining did detect all previously known RTD patients in the hospital.

**Conclusions:**

By screening a large cohort of patients of all ages in a tertiary referral hospital in a period spanning 17 years, no new RTD patients were found. Although not all suspected patients underwent genetic testing, our findings suggest that the prevalence of RTD is low and the chance of having missed this diagnosis in a tertiary referral hospital is limited.

## Background

Riboflavin transporter deficiency (RTD, OMIM 614707), an inborn error of riboflavin (vitamin B2) transport formerly known as Brown-Vialetto-Van Laere (BVVL) or Fazio Londe syndrome (OMIM 211530; 211500), causes multifocal and progressive neurological deficits.

Since 2010 it is known that biallelic variants in *SLC52A2* and *SLC52A3,* coding for the riboflavin transporter type 2 and 3 respectively, underlie this neurodegenerative disorder which was subsequently found to be successfully treatable with high dose oral riboflavin [1–3].

Patients present with multiple cranial neuropathies, with hearing loss as the most frequent symptom. Visual loss, facial weakness and marked bulbar dysfunction with difficulties speaking and swallowing may all develop over time. In addition, a peripheral neuropathy leads to limb muscle weakness, sensory ataxia and respiratory insufficiency due to weakness of the diaphragm [4, 6].

Most patients present at a young age and the disease course in infancy is rapidly progressive and lethal when left untreated. However, onset of symptoms, often with a slower rate of progression, can occur later in life, well into adulthood [4] Low plasma flavin levels, plasma acylcarnitine profiles and urine organic acid analysis are abnormal in 50% of RTD patients [4, 6]. A definitive diagnosis can only be made by identifying causative genetic variants in *SLC52A2* and *SLC52A3.* Timely treatment with high dose oral riboflavin halts disease progression and can be lifesaving [4–7].

Since 2010, an increasing number of genetically confirmed patients is reported in the literature [6] The prevalence of RTD is unknown. It is estimated that RTD affects one in 1,000,000 people in the general population [8] However since diagnostic capabilities have improved with the advent of molecular genetic testing, RTD might be more prevalent than previously thought. While the phenotypic spectrum is expanding with increasing access to genetic diagnostics, RTD may still be underdiagnosed or misdiagnosed in adults. Recognizing RTD can be challenging due to the rarity of this disease, lack of awareness of the milder end of the disease spectrum and overlap of symptoms with more common, acquired disorders, like inflammatory neuropathies. As treatment with oral riboflavin is highly effective in halting RTD progression in patients of all ages, a correct diagnosis is of utmost importance.

The aim of this retrospective datamining study was to determine the number of undiagnosed RTD patients in a large population of patients in a tertiary referral hospital.

## Methods

### Patients and study design

A systematic search in Electronic Health Records (EHR) of patients of all ages visiting the Amsterdam University Medical Centers between January 2004 and July 2021 was performed by the medical data text mining tool CT cue (CTcue text-mining software (v3.1.0, CTcue B.V., Amsterdam, The Netherlands). CT cue is a natural language processing and text mining technique, built for medical professionals, that can be used as an advanced method of information extraction of free text data in EHRs. By using a designed query, only relevant parts of the EHRs are shown and results are directly collected into a dataset [9]. Patient records, matching pre-defined search terms (see below), including Dutch and English synonyms, were selected for inclusion.

Selected EHR were pseudonymized and analyzed by two independent researchers (B.J., M.G). Patients without a diagnosis according to the information in the EHR and with symptoms and signs indicating the possibility of RTD were included. Patients, for whom a diagnosis (other than RTD) was reported in the EHR, were excluded. All included patients and patients for whom no consensus was reached between the independent researchers were discussed by the research team (B.J., M.G, A.B.).

The included patients that were alive at the time of the study were contacted through their treating physicians and invited to participate in the study. Participating patients were offered genetic testing for RTD by their treating physicians. All patients that underwent genetic testing gave written informed consent to share the results with the study team. The Medical ethics committee of the AMC provided a waiver for this study.

The search strategy for the CT Cue was developed to reflect the most frequently reported symptoms in genetically proven RTD. The search term “Hearing loss” or “Auditory neuropathy spectrum disorder” as the most frequent symptom was combined with other key clinical symptoms, or with “riboflavin”. Riboflavin was considered a relevant search term because the most frequent biochemical abnormality in RTD is an abnormal acylcarnitine profile indicative of riboflavin deficiency. Furthermore, patients may have been prescribed oral riboflavin because of the suspicion of an nonspecific mitochondrial disorder. The combination of the following search terms was used to identify all potential RTD patients:

‘Auditory neuropathy spectrum disorder’ *or* ‘hearing loss and ‘riboflavin’ *or* ‘cranial neuropathy’ *or* ‘bulbar palsy’ *or* ‘muscle weakness’ *or* ‘ataxia’.

## Results

**Fig. 1:**
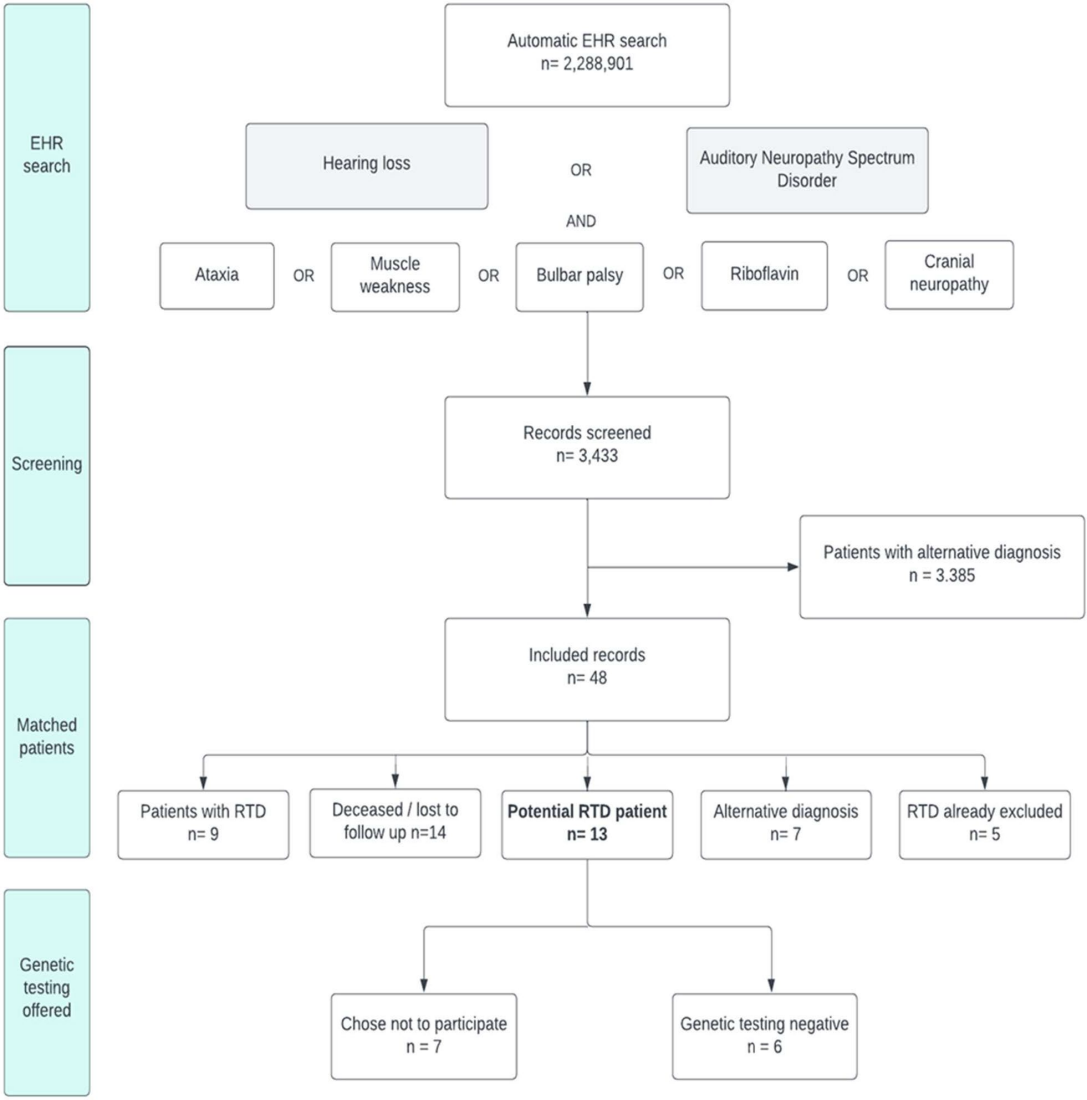
Flowchart RTD screening results

An overview of results is presented in Fig. 1. A total of 2,288,901 EHR, of all patients who visited the Amsterdam UMC’s between January 2004 and July 2021, were automatically screened based on the search terms. This identified 3,433 records which complied with the search criteria. Manual analysis of documented signs and symptoms in these pseudonymized EHR yielded 48 patients with symptoms and signs indicating the possibility of RTD without a documented alternative diagnosis. Ten patients were deceased at the time of the analysis. The age of the deceased patients ranged from 3 to 86 years (median: 68,5 years; SD: 25,3). Four patients were lost to follow up. One patient especially, with deafness, loss of vision, dysphagia and muscle weakness had a high suspicion of RTD. As she had moved abroad, we were unable to trace her.

An alternative diagnosis that explained the clinical symptoms was reported in 7 patients. The reported diagnoses were Perrault syndrome, Bardet-Biedl syndrome, hypercalcemia and different types of hereditary motor and sensory neuropathy (HMSN). In 5 patients RTD had already been ruled out by genetic testing in the past and 9 patients were already diagnosed with RTD.

Thirteen patients with a possible diagnosis of RTD were identified. After contacting the treating physicians of these patients, genetic testing was performed in six patients. Seven patients chose not to participate. Genetic evaluation did not demonstrate RTD in the six tested patients.

## Discussion

In this retrospective data mining study, we aimed to identify undiagnosed patients with RTD by screening EHR’s of all patients visiting a large tertiary referral hospital in a period spanning 17 years. Screening a total of 2,288,901 pseudonymized EHRs yielded 48 patients with possible RTD, of whom nine were known RTD patients and five had been previously genetically tested for RTD with negative results. We could not follow up ten deceased patients and four patients were lost for follow up, among whom one with a very high suspicion of RTD who had moved out of the country. All other patients alive (n = 13) were invited for genetic testing for RTD. In six patients, RTD was ruled out by genetic testing. No new patients with RTD were diagnosed.

We chose to screen with search criteria based on the most prevalent symptoms of RTD.

With this combination of search criteria, we were able to find all patients with RTD treated in our hospital during that period. However, by choosing hearing loss as a prerequisite for inclusion, some patients with RTD might not have been identified, as the vast majority, but not all patients with RTD develop hearing difficulties during the course of their disease. In different reviews on RTD the prevalence of hearing loss varied between 85–89% for RTD2 patients and 76–82% for RTD3 patients [4, 6]. It is also possible that patients with RTD that were misdiagnosed were erroneously excluded based on the reported diagnosis in their EHR.

Another limitation stems from the retrospective nature of this study, relying on the adequate documentation in the EHR of all signs and symptoms of patients.

From the selected records almost a third of the patients had died or were lost to follow up and more than half of the patients with potential RTD were not willing to participate. From the patients that died seven patients were older than 50 years old at the time of death, which makes it unlikely that they suffered from RTD. The other three patients died at 3, 36 and 45 years of age of an unknown cause.

One patient with high suspicion of RTD was lost to follow up. Therefore, we cannot rule out that we missed one or more patients with RTD, also in the group of deceased patients.

In this era where genetic testing is readily available in many hospitals, we feel that the chance of not identifying or misdiagnosing a young child with the classical and rapidly progressive symptoms of RTD is low. However, in adult patients, symptoms are less specific and disease course is usually much slower, carrying a risk of a diagnostic delay. Although a change in diet may cause a rapid deterioration in previously asymptomatic individuals [9], many adults have had an extended diagnostic journey before diagnosis. Fortunately, clinical whole-exome sequencing (WES) has dramatically increased the diagnostic yield in these patients as illustrated by the growing number of published adult RTD patients. Treatment with oral riboflavin is just as important in older patients to prevent disease progression resulting in long-term morbidity or even mortality as it is in infants [10]. It is therefore vital to be aware of their key clinical features to ensure patients are identified early, timely diagnosed and offered appropriate treatment.

Epidemiological data on the prevalence of RTD are lacking, as are carrier frequencies for the known RTD variants. Although we were unable to test all patients with symptoms indicating RTD as a possible diagnosis, our study suggests that the prevalence of undiagnosed RTD in the adult patient population is low and that at this time the chance for patients with RTD being unidentified or misdiagnosed in a tertiary referral center is limited.

## Data Availability

Data cannot openly be shared due to the need to protect the patients privacy. Data may only be provided upon reasonable request to the corresponding author.
